# Feasibility of a Home-Based Tablet App for Dexterity Training in Multiple Sclerosis: Usability Study

**DOI:** 10.2196/18204

**Published:** 2020-06-09

**Authors:** Judith Jantine Willemijn van Beek, Erwin Everardus Henri van Wegen, Marc Berend Rietberg, Thomas Nyffeler, Stephan Bohlhalter, Christian Philipp Kamm, Tobias Nef, Tim Vanbellingen

**Affiliations:** 1 Neurocenter Lucerne Cantonal Hospital Lucerne Switzerland; 2 Gerontechnology and Rehabilitation Group University of Bern Bern Switzerland; 3 Department of Rehabilitation Medicine, Amsterdam Movement Sciences Multiple Sclerosis Center Amsterdam, Amsterdam University Medical Centers Vrije Universiteit Medical Center Amsterdam Netherlands; 4 Department of Neurology Bern University Hospital Bern Switzerland

**Keywords:** dexterity, feasibility, multiple sclerosis, rehabilitation, app, home-based training

## Abstract

**Background:**

Persons with multiple sclerosis (MS) often experience dexterous difficulties during the performance of activities of daily living, such as fastening buttons, handling coins, or writing, therefore impacting their health-related quality of life. Mobile health (mHealth) solutions, such as tablet apps, may be used to train impaired dexterous skills. The feasibility of a tablet app–based dexterity home-based intervention in MS (TAD-MS) has not been explored yet in persons with MS.

**Objective:**

The aim of this pilot study was to evaluate the feasibility and usability of home-based dexterity training with a tablet app in both persons with MS and healthy subjects.

**Methods:**

A total of 9 persons with MS, aged 35-71 years, with an Expanded Disability Status Scale score between 2 and 7.5, performed the TAD-MS for 4 weeks, five times a week, with each training session lasting approximately 30 minutes. Participants’ impaired dexterity was measured by the Nine-Hole Peg Test. A total of 10 age-matched healthy subjects also tested and rated the usability of the app. Outcome measures were the adherence rate as well as usability measured by the System Usability Scale and a Custom User Engagement Questionnaire (CUEQ).

**Results:**

High feasibility of the tablet app–based dexterity training program was shown by a 97% adherence rate to the training protocol (ie, mean 19.4/20 sessions completed, SD 0.8). High system usability scores (ie, mean 85.39%, SD 11.67) and overall high scores given in the CUEQ (ie, mean 8.2/10, SD 1.4) further point to high usability of the app. Neither demographic variables nor dexterity levels affected the use of the app.

**Conclusions:**

This pilot study is the first to demonstrate high feasibility and usability of a new tablet app–based dexterity home-based training program among both persons with MS and healthy individuals. Whether this kind of training improves dexterity will need to be evaluated in a randomized controlled trial.

## Introduction

Multiple sclerosis (MS) is the most common cause of nontraumatic neurological disability in young adults and comes with long-term disability, including dexterous dysfunction[1-3]. Impaired dexterity is a frequently reported problem in MS, affecting approximately 70%-80% of persons with MS [4], mainly due to deconditioning, coordination deficits, muscle weakness, and/or tremor [5]. Subsequently, persons with MS often experience problems in performing activities of daily living (ADL), such as fastening buttons, handling coins, or writing, therefore negatively impacting their self-reported health-related quality of life [6].

In the last decade, the implementation of technical innovations has rapidly grown within MS neurorehabilitation. Many of these innovations relate to mobile health (mHealth) solutions and are often *apps* that can easily be installed on smartphones and/or tablets [7] for use in the home environment. Their use is increasing among today’s MS population [7]. Recently, Marrie and colleagues showed in a survey, which included over 6000 persons with MS, that around 84% already used mHealth services for exchanging medical information [8]. Approximately half of the smartphone and tablet users used an mHealth app. Interestingly, a large majority of users (98.7%) reported some kind of benefit (eg, increased physical activity or being better informed about diagnosis) [8].

The most frequently included features in such apps focus on personal data management and patient education (ie, health status) by providing, for example, diaries [9,10]. Some apps directly tackle physical or cognitive performance, such as the Cognitive Training Kit (COGNI-TRAcK) app [11]. This app was developed to train cognitive deficits, which are common among persons with MS [12,13]. COGNI-TRAcK showed high feasibility and usability as well as improved working memory if the app was used within a home-based cognitive training environment [14]. The home-based training environment certainly added to the feasibility, because the persons with MS were flexible in planning their sessions and saved on travel time to rehabilitation centers. The specific contents of the app were also experienced to mentally stimulate the users, which helped to sustain motivation, and the contents were fun to play, explaining the high adherence to the training protocol [14].

With regard to physical performance, Thirumalai and colleagues recently developed the therapeutic exercise TEAMS (Tele-Exercise and Multiple Sclerosis) app, aiming to be a comprehensive home tele-exercise program [15]. It contains several exercise videos and articles, explaining yoga, Pilates, and dual-tasking [15]. The advantage of this app is that the physical activity of persons with MS at home can be enhanced through tele-exercising. However, the primary aim of this app was to promote overall physical activity and not dexterity. So far, one app to measure dexterity in MS [16] and two dexterity training apps [17,18] have been developed and tested for their feasibility, either among stroke survivors [17] or among people after surgical carpal tunnel release [18]. The content of these training apps was primarily based on tapping or pinching performance of single digits [17,18]. An advantage of both apps was that finger movements, performed while touching the touchscreen, could be guided by feedback. To our knowledge, the usability of a training dexterity app has not been explored yet in persons with MS.

This pilot study comprehensively evaluated the feasibility and usability of a new tablet app–based dexterity home-based intervention in MS (TAD-MS) [6] in both persons with MS as well as healthy subjects. The exercises focus on various components of dexterity, such as the pinch grip, coordinated finger movements, and tapping movements, and are designed to be challenging and fun. We hypothesized that the new dexterity app is feasible and usable, for both persons with MS and healthy individuals. Furthermore, we expected high adherence to the home-based training protocol.

## Methods

### Participants and Setting

For this pilot study, both persons with MS and healthy subjects participated. Patients were prospectively recruited from the Neurocenter, Lucerne Cantonal Hospital, Lucerne, Switzerland. They were included if they met the following inclusion criteria: (1) provided written informed consent according to the latest Declaration of Helsinki [19], (2) had an MS diagnosis following the revised McDonald criteria (ie, primary or secondary progressive or relapsing-remitting MS) [20], (3) experienced impaired dexterity in their ADL or had a pathological Nine-Hole Peg Test (9HPT) according to established cutoff values [21], (4) aged above 18 years, and (5) were able to understand German for instructions and measurements. Patients were excluded if they (1) suffered from severe cognitive impairment (ie, Montreal Cognitive Assessment score less than 21 [22]) or (2) had other neurological, psychiatric, or developmental diseases prior to MS diagnosis. The Ethics Committee of Northwest and Central Switzerland (EKNZ) approved this study. We aimed to recruit age- and gender-matched healthy subjects, who worked as health care professionals or other staff members at the cantonal hospital, to explore whether usability scores of the TAD-MS differed between persons with MS and healthy individuals.

### Tablet App–Based Dexterity Training

For eligible participants, an intake meeting in our clinic was planned. Upon consent, descriptive characteristics were assessed and participants were asked whether they were having other treatment sessions (ie, physiotherapy or occupational therapy). If *yes*, all subjects were allowed to participate if their regime was not related to dexterity, since that would have interfered with the outcome of this study. Subsequently, all patients received instructions about the TAD-MS by an expert clinician (JJWvB). The intervention consisted of five 30-minute sessions per week for 4 weeks; therefore, in total, 20 home sessions were completed. Patients received a tablet with the installed app and a personalized log-in code. An Apple iPad (Manufacturer Part Number: MP2G2TY; 9.7-inch Retina display; Wi-Fi; 2017) or Samsung Galaxy Tab S2 (model: SM-T813; 9.7-inch display; Wi-Fi) was used. The app was developed by a team of experts, consisting of an occupational therapist (Amy Orellana, see Acknowledgments), two graphic designers, and one programmer. In line with the Technology Acceptance Model, which is a frequently used acceptance framework for understanding clinicians’ adoption of mHealth [23], perceived usefulness, perceived ease of use, and enjoyment were key elements that were integrated into this app. The programmer wrote the software program for the app with the programming language C# Job System using Unity 5.0 (Unity Technologies) [24]. The team met on a weekly basis to test and retest the function of the exercises, to resolve bugs, and to review the progress of the software.

**Figure 1:**
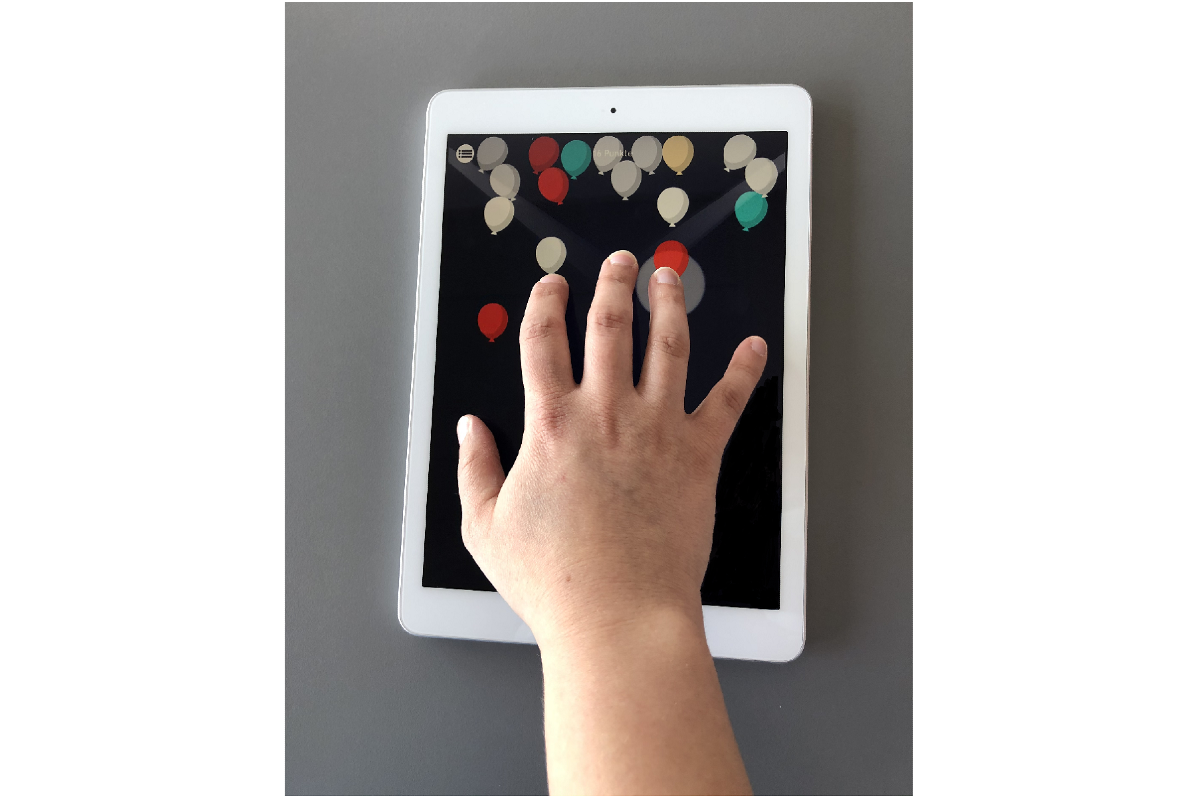
The TAD-MS app contains six dexterity exercises, including selective finger tapping (balloons), which is shown here. Other exercises are acrobat seesaw (pinch grip), the wheel (rotation and flexion/extension in various joints in fingers), and elevator I (selective finger rotation). TAD-MS: tablet app–based dexterity home-based intervention in multiple sclerosis.

Besides an explanation of the exercises from the clinician (JJWvB), the participant got instructions through instructional videos and texts for each exercise separately in the app, explaining how to perform the exercise. An example of the exercises is shown in Figure 1. For a detailed description of the exercises, we refer to our protocol paper where we comprehensively explained the six dexterous games [6]. The clinician (JJWvB) checked the adherence to the protocol by logging in to a website where the performance of the participants was tracked and recorded.

The healthy subjects were invited to our clinic, and after receiving instructions by a trained clinician (Cleo Bol, see Acknowledgments), they performed all the dexterous exercises in a single session. Both patients and healthy subjects did all the exercises with both hands separately, starting with their dominant hand. Patients could choose for themselves when to do the exercises (ie, morning, afternoon, or evening) but were instructed to not train twice on the same day.

### Objectives

Our first objective was to extensively evaluate the feasibility and usability of a specific tablet app–based dexterity home-based training program in both persons with MS and healthy subjects. Our second objective was to assess the adherence to the protocol. Our third objective was to investigate whether demographic variables (ie, age and disease duration, type, and severity) and dexterous abilities could interfere with the use of the app.

### Endpoints

All measurements were collected and rated by a single expert clinician (JJWvB) to achieve an optimal standardization of outcome measurements. The endpoints were feasibility measures: (1) the adherence to the protocol, (2) the self-reported System Usability Scale (SUS) score, and (3) a Custom User Engagement Questionnaire (CUEQ) (see Multimedia Appendix 1), where the opinions of the participants were sought, regarding the content of the app exercises as well as the home-based training.

Based on our previously published pilot study [25], the adherence rate was determined as the ratio of the *number of home sessions performed* (SP) and the *prescribed number of sessions* (PS), which is 20: SP/PS × 100% = SP/20 × 100%. An adherence rate of 80% or higher was considered as *good*.

The SUS was filled out after completing the intervention. The SUS is a well-validated questionnaire, consisting of a 10-item, 5-point Likert scale—each item was scored from 0 (*strongly disagree*) to 4 (*strongly agree*)—which takes three usability criteria into account: effectiveness, satisfaction, and efficiency [26,27]. The total score is obtained by multiplying the mean sum value by 2.5. The SUS score ranges from 0% to 100%, where a higher score indicates better system usability. A score of 70% up to a maximum of 100% represents acceptable-to-excellent usability [26,27].

The CUEQ contained two parts. Firstly, seven specific questions (Qs) related to the content of the app had to be scored on a scale ranging from 1 (*poor*) to 5 (*excellent*) (ie, Q1 = Were the exercises fun to play?; Q2 = Have you improved your fine motor skills?; Q3 = Was it easy to integrate the exercises into daily life?; Q4 = Were the explanations for the execution of each exercise sufficient?; Q5 = Would you recommend this application?; Q6 = Can you take the tablet well and easily on the go?; and Q7 = Do you notice improvements in everyday life regarding fine motor skills?). Since the healthy subjects did not participate in the training protocol, they did not answer Q2 and Q7. Secondly, all participants rated the overall quality of the app, which ranged from 1 (*very poor quality*) to 10 (*excellent quality*). Furthermore, they were asked to make suggestions for further improvement regarding the content of the app. All questions of the CUEQ were defined based on agreement between two experts in the field of dexterity rehabilitation (TV and Amy Orellana, see Acknowledgements); therefore, they were based on face validity and content validity.

Besides demographics, dexterous functions were measured in the patient cohort to get an impression of overall disability and to check whether impaired dexterity may interfere with the use of the app. We used a patient-reported outcome measure (ie, the Arm Function in Multiple Sclerosis Questionnaire [AMSQ]), a performance-based task (ie, the 9HPT), and a measure of the degree of neurologic impairment (ie, the Expanded Disability Status Score [EDSS]). The 9HPT [28] and the AMSQ [29,30] have shown good reliability and validity in MS. The AMSQ consists of 31 questions on a unidimensional scale that are formulated as “During the past two weeks, to what extent has MS limited your ability to...?’’ with a 6-point response option ranging from 1 (*not at all limited*) to 6 (*no longer able to*). One final score is obtained, ranging from 31 to 186, with a higher score indicating more difficulties in dexterous function [29,30]. For the 9HPT, the time to perform the task was measured in seconds for each hand separately. Normative values considering age and gender are available [21]. The EDSS examines neurological symptoms and signs in eight functional systems. Scores range from 0 (*normal neurological examination)* to 10 (*death due to MS)* in 0.5-unit increments that represent higher levels of disability [31].

### Statistical Analyses

Descriptive statistics were used to present the baseline and clinical characteristics of all participants. The observed data approach was used for missing data. The normality of distribution of all outcome measurements was checked by Shapiro-Wilk tests. Moreover, skewness and kurtosis of the residuals were used to further check for normal distribution of outcome scores. To explore relationships between clinical parameters (ie, age, gender, disease type, disease severity, disease duration, AMSQ score, and 9HPT time) and usability scores, Spearman or eta correlations were performed.

The level of significance was set at *P*≤.05 (two-tailed). Statistical analyses were performed using SPSS Statistics for Windows, version 26.0 (IBM Corp).

## Results

Recruitment took place between April 2018 and May 2019. A total of 9 persons with MS started and completed the intervention. No adverse effects or complications and no dropouts were reported. In addition, 10 age-matched healthy subjects participated (mean age 52 years, SD 13, range 33-73). Detailed clinical and demographic characteristics of the persons with MS are presented in Table 1.

The adherence to the training protocol by the persons with MS with regard to the average total sessions completed was very high. More specifically, the persons with MS completed, on average, 19.4 sessions (SD 0.8) of the planned 20 sessions (97%).

**Figure 2:**
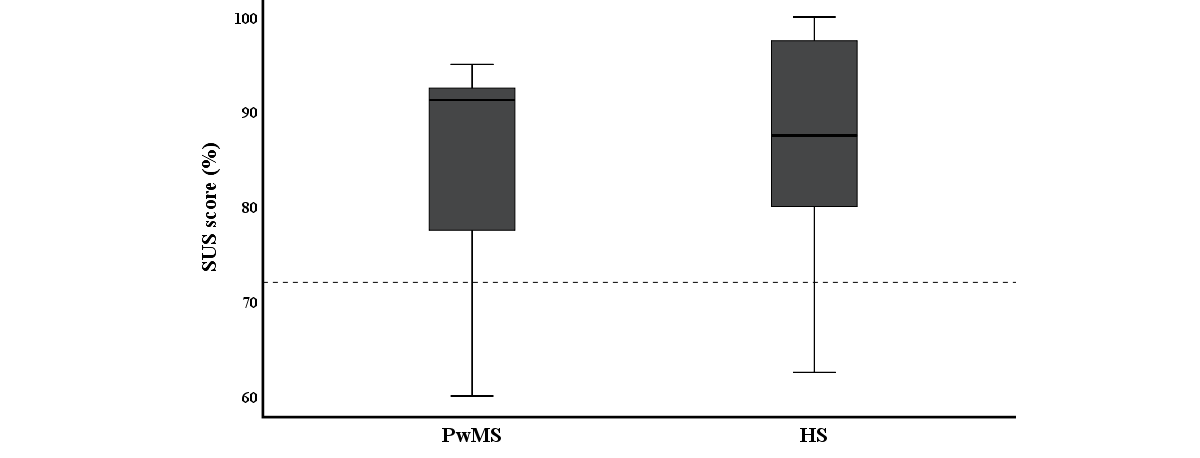
Results of the System Usability Scale (SUS). The SUS score ranges from 0% to 100%, where a higher value indicates better system usability. The dotted line represents a score of 71.4%. A score of 70% up to a maximum 100% represents acceptable-to-excellent usability. PwMS: persons with MS; HS: healthy subjects.

Regarding the self-reported system usability of the app, the mean SUS scores were 85.00% (SD 11.59) and 85.75% (SD 12.36) for the patients and the healthy subjects, respectively, indicating excellent usability (see Figure 2). There was no significant difference between these scores (Mann-Whitney test, *P*=.74). Out of 10 healthy subjects, 2 (20%) rated the system just below the cutoff of 71.4%, meaning acceptable usability. Out of 9 patients, 1 (11%) rated the app as 60%, which means moderate usability. The overall SUS score (N=19) was 85.39% (SD 11.67).

**Figure 3:**
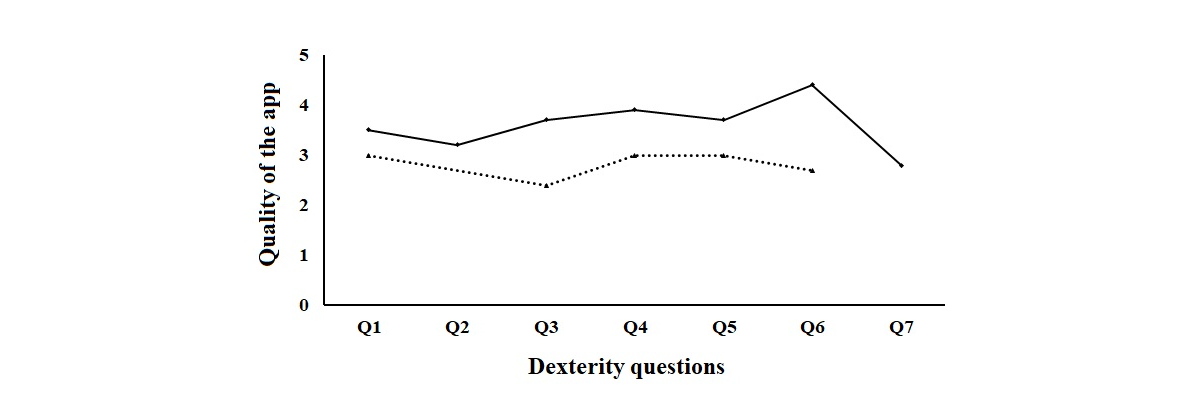
Quality ratings of the app regarding dexterity-related questions (Qs). The continuous line represents persons with multiple sclerosis. The dashed line represents healthy subjects. Scores range from 1 to 5: 1 = bad; 2 = moderate; 3 = good; 4 = very good; and 5 = excellent. Q1 = Were the exercises fun to play?; Q2 = Have you improved your fine motor skills?; Q3 = Was it easy to integrate the exercises into daily life?; Q4 = Were the explanations for the execution of each exercise sufficient?; Q5 = Would you recommend this application?; Q6 = Can you take the tablet well and easily on the go?; and Q7 = Do you notice improvements in everyday life regarding fine motor skills?

The CUEQ revealed high scores on each dexterity-related question, and the overall quality of the app was scored with a mean of 8.20 (SD 1.35), further indicating very good usability. Only 1 person out of 9 (11%) with MS gave it a score of 5, indicating moderate usability. The others (8/9, 89%) gave it a score of 7 or higher. None of the participants needed additional technical support at home, further denoting the usability. The mean quality scores of the dexterity-related questions for both groups are shown in Figure 3.

We did not find significant correlations between the SUS scores and age (*r*=.20, *P*=.42), gender (eta=.024), disease type (eta=.16), disease severity (*r*=–.46, *P*=.22), disease duration (*r*=.34, *P*=.38), AMSQ score (*r*=–.59, *P*=.09), and 9HPT time (*r*=–.59, *P*=.10).

**Table 1:** Clinical and demographic characteristics of the persons with multiple sclerosis (MS).

Characteristic		Value (n=9)	
Age in years, mean (SD); range		53.89 (12.27); 35-71	
**Gender, n (%)**			
	Male		0 (0)
	Female		9 (100)
**Handedness, n (%)**			
	Right		9 (100)
	Left		0 (0)
**MS type, n (%)**			
	Relapsing-remitting		5 (56)
	Primary progressive		2 (22)
	Secondary progressive		2 (22)
EDSS^a^ score, mean (SD); range		3.89 (1.95); 2.0-7.5	
Disease duration in years, mean (SD); range		10.56 (9.76); 1-28	
AMSQ^b^ score, mean (SD); range		53.78 (21.38); 36-99	
**9HPT^c^ in seconds, mean (SD); range**			
	Right + left		26.27 (8.71); 20.20-46.87
	Right		24.17 (5.20); 18.67-35.50
	Left		28.36 (14.60); 19.51-65.53

^a^EDSS: Expanded Disability Status Scale. Scores range from 0 (normal neurological examination) to 10 (death due to MS) in 0.5-unit increments that represent higher levels of disability.

^b^AMSQ: Arm Function in Multiple Sclerosis Questionnaire. Scores, for each of the 31 questions, range from 1 (not at all limited) to 6 (no longer able to), with total scores ranging from 31 to 186.

^c^9HPT: Nine-Hole Peg Test.

## Discussion

### Principal Findings

We extensively evaluated the feasibility and usability of a tablet app–based dexterity home-based training program in both persons with MS and healthy subjects. The very high adherence rate toward the training protocol, being 97%, points to the excellent feasibility of the TAD-MS, which is further supported by the absence of dropouts. Regarding usability, both persons with MS as well as healthy subjects scored high on the SUS, were very satisfied with the system features of the app, and highly appreciated its easy integration into their daily life setting. Furthermore, our findings suggest that demographic factors (ie, gender; age; and disease duration, type, and severity) and dexterity levels do not interfere with the use of the app, further adding to the high usability of this mHealth intervention.

In contrast with the only two previous studies that tested the use of different dexterity apps in other populations, which were stroke survivors [17] and patients suffering from carpal tunnel syndrome [18], our pilot study shows for the first time a high adherence rate for a 4-week, tablet app–based dexterity training program in persons with MS. The duration and dosage of our training protocol, the TAD-MS, actually matched those from a previous dexterity app study [18] and is also in line with other home-based dexterity training programs in MS [32,33], which showed similar adherence rates. The fact that all included subjects did not need additional technical support at home, were independent, and were free to choose when to do their exercises (ie, morning, afternoon, or evening) likely contributed to the high adherence.

The user interface of this app was simple and clear, mainly explaining the high usability scores. Previous recent reviews [9,10] and surveys [7,8,34] about mHealth apps for MS already suggested some key features for the development of user-friendly apps. These features were as follows: easy app navigation, clear instructions of the exercises, aspects of gamification, and low technical requirements. This app fulfilled these key elements, since persons with MS could easily navigate through the app, with each exercise having clear instructions (ie, video sequences demonstrating and explaining the aim of the exercises). In addition, gamification elements and integrated feedback to optimize performance were incorporated, both aspects that are important to build and maintain motivation during training, as suggested before [9,34].

Regarding the effect of demographic variables on app usability scores, we did not find an association between the user interface and demographic variables, supporting the generalizability of the approach and indicating that the app is suitable for a broad MS population. This finding is in line with a previous report that found that irrespective of age, disease type, and disease severity, persons with MS were able to manage technical issues of a new mHealth app [35]. However, our results do contrast with Kizony and colleagues [17], who showed that declines in age-related dexterity, as previously shown in healthy subjects [36,37], could affect app performance. Thus, older healthy subjects were more challenged to use the app properly. The reason why we could not find such a relationship, besides the limited sample size, may lie in the heterogeneity of the MS disease, meaning that both younger and older patients, being differently affected by the disease, may have similar experiences using our app. The level of impaired dexterity, irrespective of age, might be a bigger challenge. In MS, a possible impact of impaired fine motor skills, mainly due to underlying ataxia, muscle weakness, and/or tremor [2], on app usability has been suggested as a possible barrier [7,34]. However, in our study, we could not find a relationship between validated dexterity measures and usability scores for this app, suggesting this might not be such a decisive barrier.

### Limitations

This pilot study does have some limitations. We included a small sample of participants with relatively good cognitive function. Therefore, we cannot generalize our findings to individuals suffering from severe cognitive disabilities. However, this was not the scope of this pilot study. For persons with MS with severe cognitive impairment, more conventional dexterity-training methods might be more useful [32], because real objects and tools are used within this kind of training, certainly being more closely related to real dexterity ADL. Finally, although we included a healthy control group, these subjects did not have any dexterous impairment and, therefore, did not participate in the 4-week training. Future studies could explore whether healthy older subjects with some dexterous disability could benefit from the TAD-MS.

### Conclusions

This pilot study is the first to demonstrate the high feasibility and usability of a new tablet app–based dexterity home-based training program, called the TAD-MS [6], among both persons with MS and healthy subjects. The tablet app training program can be easily integrated into the home situation, without any technical or therapeutic support, therefore providing an additional tool in the rehabilitation toolbox to train dexterity for persons with MS. Whether the TAD-MS improves dexterity will need to be assessed in future studies (ie, in a pre-post design) that can provide effect sizes that are necessary to perform appropriate sample size calculations for randomized trials.

## Appendix

Multimedia Appendix 1 Custom User Engagement Questionnaire.

## Abbreviations

9HPT: Nine-Hole Peg Test
ADL: activities of daily living
AMSQ: Arm Function in Multiple Sclerosis Questionnaire
COGNI-TRAcK: Cognitive Training Kit
CUEQ: Custom User Engagement Questionnaire
EDSS: Expanded Disability Status Score
EKNZ: Northwest and Central Switzerland
mHealth: mobile health
MS: multiple sclerosis
PS: prescribed number of sessions
Q: question
SP: number of home sessions performed
SUS: System Usability Scale
TAD-MS: tablet app–based dexterity home-based intervention in multiple sclerosis
TEAMS: Tele-Exercise and Multiple Sclerosis
