# Supplementary material for: Feasibility of a Home-Based Tablet App for Dexterity Training in Multiple Sclerosis: Usability Study
Source: JMIR Mhealth Uhealth. 2020 Jun 9;8(6):e18204. doi: 10.2196/18204 (PMC7312262; doi:10.2196/18204)
Supplement: Multimedia Appendix 1 [file mhealth_v8i6e18204_app1.pdf]

Custom User Engagement Questionnaire

Pat. ID: ______________________________

Date: ______________________________

Thank you for your evaluation

1. Content of the app

	Poor	Fair	Good	Very good	Excellent
1. Were the exercises fun to play?					
2. Have you improved your fine motor skills?					
3. Was it easy to integrate the exercises into daily life?					
4. Were the explanations for the execution of each exercise sufficient?					
5. Would you recommend this application?					
6. Can you take the tablet well and easily on the go?					
7. Do you notice improvements in everyday life regarding fine motor skills?					

2. Overall score

(please tick one number)

Very poor	1	2	3	4	5	6	7	8	9	10	Excellent

Do you have any suggestions for improvement, further ideas for exercises?

____

____

Further comments:

____

____

Amy Orellana & PD Dr. Tim Vanbellingen
